# Binge ethanol exposure causes endoplasmic reticulum stress, oxidative stress and tissue injury in the pancreas

**DOI:** 10.18632/oncotarget.11103

**Published:** 2016-08-05

**Authors:** Zhenhua Ren, Xin Wang, Mei Xu, Fanmuyi Yang, Jacqueline A. Frank, Zun-ji Ke, Jia Luo

**Affiliations:** ^1^ Department of Anatomy, School of Basic Medicine, Anhui Medical University, Hefei, China; ^2^ Department of Pharmacology and Nutritional Sciences, University of Kentucky College of Medicine, Lexington, Kentucky, USA; ^3^ Department of Biochemistry, Shanghai University of Traditional Chinese Medicine, Shanghai, China

**Keywords:** apoptosis, glucose, inflammation, insulin, pancreatitis, Pathology Section

## Abstract

Alcohol abuse is associated with both acute and chronic pancreatitis. Repeated episodes of acute pancreatitis or pancreatic injury may result in chronic pancreatitis. We investigated ethanol-induced pancreatic injury using a mouse model of binge ethanol exposure. Male C57BL/6 mice were exposed to ethanol intragastrically (5 g/kg, 25% ethanol w/v) daily for 10 days. Binge ethanol exposure caused pathological changes in pancreas demonstrated by tissue edema, acinar atrophy and moderate fibrosis. Ethanol caused both apoptotic and necrotic cell death which was demonstrated by the increase in active caspase-3, caspase-8, cleaved PARP, cleaved CK-18 and the secretion of high mobility group protein B1 (HMGB1). Ethanol altered the function of the pancreas which was indicated by altered levels of alpha-amylase, glucose and insulin. Ethanol exposure stimulated cell proliferation in the acini, suggesting an acinar regeneration. Ethanol caused pancreatic inflammation which was indicated by the induction of TNF-alpha, IL-1beta, IL-6, MCP-1 and CCR2, and the increase of CD68 positive macrophages in the pancreas. Ethanol-induced endoplasmic reticulum stress was demonstrated by a significant increase in ATF6, CHOP, and the phosphorylation of PERK and eiF-2alpha. In addition, ethanol increased protein oxidation, lipid peroxidation and the expression of iNOS, indicating oxidative stress. Therefore, this paradigm of binge ethanol exposure caused a spectrum of tissue injury and cellular stress to the pancreas, offering a good model to study alcoholic pancreatitis.

## INTRODUCTION

The pancreas is an important organ responsible for glucose homeostasis and the digestion of carbohydrates, proteins and lipids. When the pancreas becomes inflamed, its digestive enzymes leak out and attack the pancreas itself as well as its surrounding organs. The inflammation of the pancreas is called pancreatitis which is a serious public health concern. There are two forms of pancreatitis: acute and chronic pancreatitis. Acute pancreatitis (AP) occurs when the pancreas suddenly becomes inflamed. AP is characterized by local and systemic inflammation which is mediated by inflammatory cytokines/chemokines and damages to acinar cells in the exocrine pancreas. AP improves and recovers as the inflammation eases. Unfortunately, approximately 20% of AP progresses to severe acute pancreatitis (SAP), a disease with high morbidity and mortality [1, 2]. AP is the most common gastrointestinal disease requiring hospitalization in the United States [2, 3]. In 2009, there were 275,000 admissions for AP, accounting for a direct annual cost of $2.6 billion [3]. Chronic pancreatitis (CP) is a progressive inflammatory disease leading to irreversible destruction of the pancreas. It is characterized by persistent inflammation, the development of fibrotic scarring and the loss of pancreatic function. CP is manifested by a spectrum of clinical symptoms ranging from severe pain to maldigestion and diabetes. It is generally believed that AP and CP are related and repeated episodes of AP could result in CP [2]. The progression of AP to CP is associated with the frequency and severity of the acute attacks [4]. Therefore, the mechanisms underlying the initiation of AP and CP are likely similar.

Alcohol abuse is associated with the development of both AP and CP [5–8]. Alcoholic pancreatitis represents 36% of all cases of AP [9]. Five percent of alcoholics develop AP [10]. Pancreatitis is the most common alcohol-related hospital diagnosis in the United States [11]. The prevalence of alcoholic pancreatitis may be much higher than the current estimation. A postmortem study showed that pancreatitis was found in up to 75% of alcoholics although clinical pancreatitis is only diagnosed in less than 10% of alcoholic patients [12, 13]. It is suggested that alcoholic AP and CP are the same disease at different stages [14]. Notably, after a first acute episode of pancreatitis, alcoholics have a much higher risk of developing CP than non-drinkers or occasional drinkers [15].

Binge drinking (episodic heavy alcohol consumption) is also an epidemic that has continued to worsen over the past decade [16, 17]. Binge drinking over a short period imposes a higher risk for pancreatitis than a moderate drinking over an extended period [18]. A single episode of binge drinking may be sufficient to induce AP [19]. The mechanisms underlying the development of alcoholic AP are unclear. This study evaluated binge ethanol exposure-induced pancreatic injury. We show here that binge ethanol exposure caused a spectrum of pancreatic injury and inflammation characteristic of AP. It also induced endoplasmic reticulum stress and oxidative stress in the pancreas, and therefore offers a good model system to investigate alcoholic pancreatitis, particularly alcohol induced AP.

## RESULTS

### Binge ethanol exposure causes pancreatic injury

**Figure 1:**
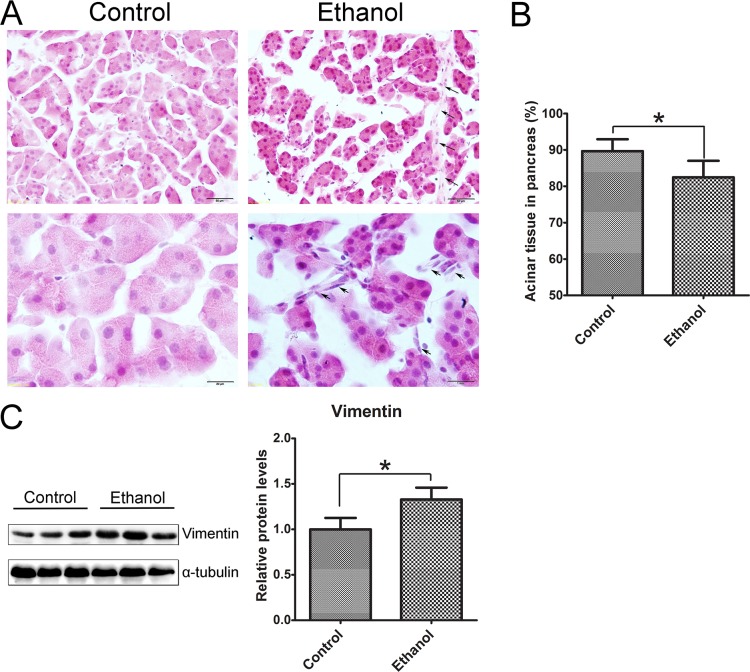
Binge ethanol exposure-induced histological alterations in the pancreas Mice were exposed to binge ethanol for 10 days as described in the Materials and Methods. Six hours after last ethanol exposure, mice were euthanized and the pancreas was processed for H&E staining and immunoblotting analysis. **A.** Representative images of H&E-stained pancreatic tissue are shown. Arrows indicate the spindle-shaped cells in intralobular and pericellular regions of the pancreas in ethanol-treated group. Bar = 50 μm in top panel (magnification of 40X); Bar = 20 μm in bottom panel (magnification of 100X). **B.** The area occupied by acini in the pancreas was calculated with an image analysis system. The percentage of area occupied by acini in total pancreatic tissue was determined. Twenty fields at 40X were randomly selected and analyzed for each animal. **C.** The expression of vimentin in the pancreas was determined by immunoblotting. Each lane in the immunoblotting image represents one animal (panel on the left). The expression of vimentin was quantified and normalized to the expression of α-tubulin (panel on the right). Each data point was the mean ± SEM of three independent experiments. * denotes statistical difference (*p* < 0.05) from the control.

We used oral gavage (5 g/kg; 25% w/v) which produced high blood ethanol concentrations (BEC) (374-463 mg/dl) to mimic human binge drinking. We first evaluated the histological changes in the pancreas following binge ethanol exposure (Figure 1). In ethanol-treated group, the size of acinar cells was smaller compared to the control group and the space between acini was increased (Figure 1A). The overall acinar volume was significantly decreased the in ethanol-treated pancreas (Figure 1B). The changes are usually indicative of tissue edema [20, 21]. In ethanol-treated tissue, there were some spindle-shaped cells in the pericellular or intralobular areas of the pancreas (Figure 1A), which is usually indicative of fibrosis [21, 22]. Ethanol exposure also increased the expression of vimentin (Figure 1C). Enhanced vimentin expression usually indicates the activation of pancreatic stellate cells which play a key role in tissue fibrosis [2, 23].

**Figure 2:**
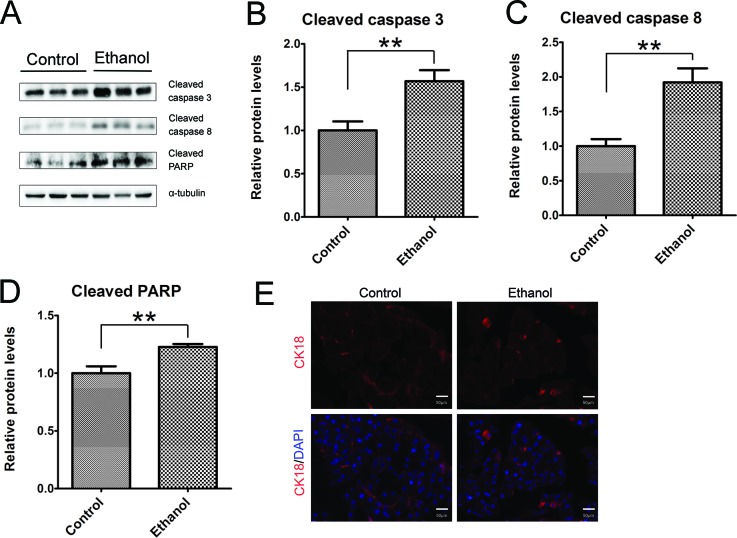
Binge ethanol exposure-induced apoptosis in the pancreas **A.** Mice were exposed to binge ethanol for 10 days as described in the Materials and Methods. Six hours after last ethanol exposure, mice were euthanized and the pancreas was processed for immunoblotting analysis of cleaved caspase-3, caspase-8 and PARP. **B.**-**D.** The expression of these proteins was quantified and normalized to the expression of α-tubulin. Each data point was the mean ± SEM of three independent experiments. ** denotes significant difference (*p* < 0.01) from the control. **E.** The apoptotic cells were determined by IHC using an anti-CK18 (caspase-cleaved product of cytokeratin 18) antibody. Bar = 50 μm (magnification of 40X).

As shown by immunoblotting data, binge ethanol exposure increased the active form (cleaved form) of caspase-3 and caspase-8 (Figures 2A-2C), suggesting that ethanol caused apoptosis in the pancreatic tissue. This was further confirmed by ethanol-induced cleavage of PARP which is a target of caspases and a marker of active apoptosis (Figure 2D). Cleaved cytokeratin 18 (CK18) which is the product of caspase-mediated cleavage has been frequently used as a marker of apoptosis. CK18-positive cells were significantly increased in the pancreatic tissues after binge ethanol exposure (Figure 2E).

**Figure 3:**
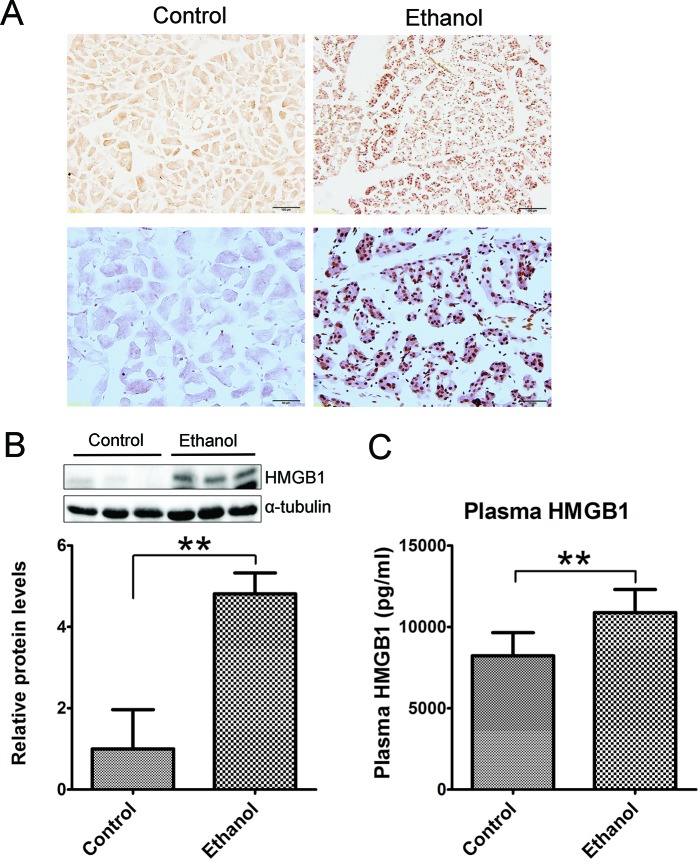
Effect of binge ethanol exposure on HMGB1 expression in the pancreas The effect of binge ethanol exposure on HMG1 in the pancreas was evaluated by immunohistochemistry (IHC) **A.** and immunoblotting **B.** Bar = 100 μm in the top panel (magnification of 20X); Bar = 50 μm in the bottom panel (magnification of 40X). HMGB1 expression was quantified and normalized to the expression of α-tubulin. The plasma levels of HMG1 were determined by ELISA **C.** Each data point was the mean ± SEM of three independent experiments. ** denotes significant difference (*p* < 0.01) from the control.

Histological analysis did not reveal apparent necrosis in the pancreas. However, immunoblotting analysis and IHC showed a drastic increase in high mobility group protein B1 (HMGB1) following binge ethanol exposure (Figures 3A and 3B). HMGB1 was originally identified as a DNA-binding protein that functioned as a structural co-factor critical for proper transcriptional regulation in somatic cells. It is released into the extracellular environment during necrosis but not apoptosis and used as a marker for necrosis [24]. In addition, the plasma HMGB1 levels were markedly increased after binge alcohol exposure (Figure 3C). The results suggested the occurrence of necrosis in the acini after binge ethanol exposure.

**Figure 4:**
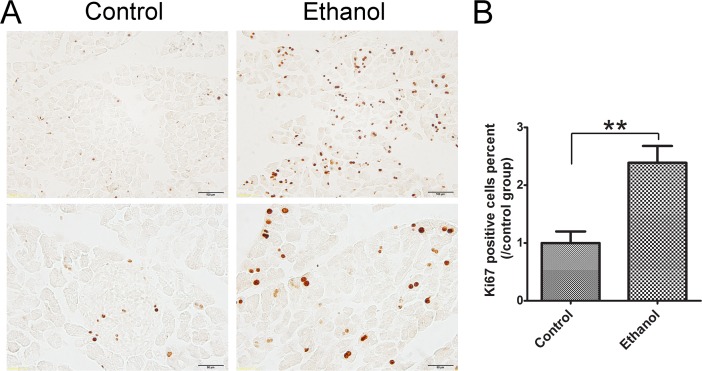
Effect of binge ethanol exposure on cell proliferation in the pancreas **A.** The proliferating cells were determined with Ki67 IHC. The representative images revealing nuclear staining of Ki67 are shown. Bar = 100 μm in the top panel (magnification of 20X) and Bar = 100 μm 50 μm in the bottom panel (magnification of 40X). **B.** Twenty fields covering at least 1,000 cells were randomly selected and Ki67 positive cells were counted at 40X magnification. Three-four animals were analyzed for each group. The percentage of Ki67 positive cells was calculated and expressed relative to the control. Each data point was the mean ± SEM of three independent experiments. ** denotes significant difference (*p* < 0.01) from the control.

Ki67 is a nuclear nonhistone protein that is universally expressed in proliferating cells and absent in quiescent cells [25]. In control tissues, there were few Ki67-positive cells in the pancreatic acini. Ethanol significantly increased the number of Ki67-positive cells in the acini (Figure 4), suggesting that acinar cells underwent a regeneration in response to ethanol-induced damage.

**Figure 5:**
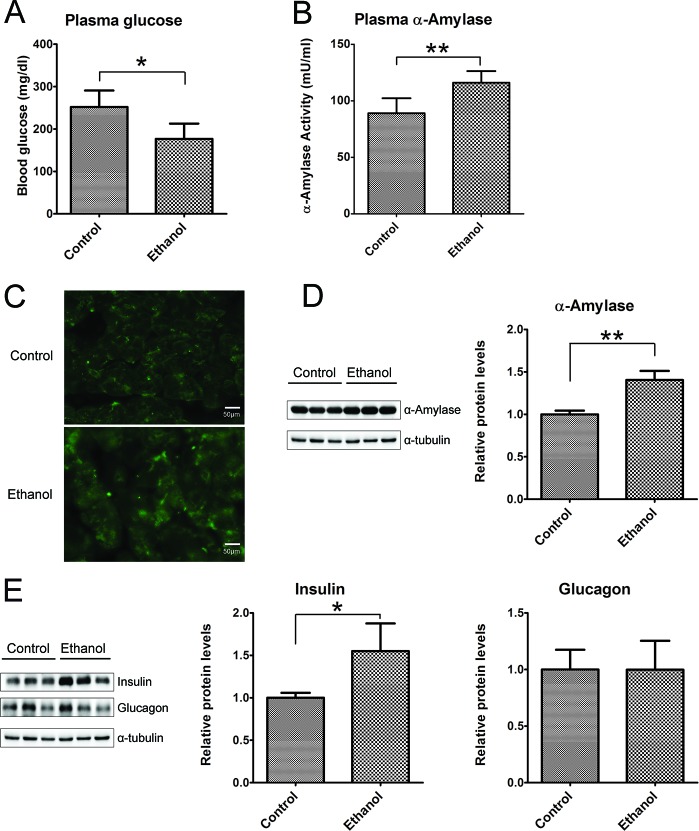
Effect of binge ethanol exposure on pancreatic function **A.** and **B.** the glucose and α-amylase levels in the plasma were determined as described in the Materials and Methods. **C.** The expression of α-amylase (green) in the pancreas was detected by immunofluorescent staining. Bar = 50 μm in the top panel (magnification of 40X). **D.** The expression of α-amylase in the pancreas was determined by immunoblotting analysis. **E.** The expression of insulin and glucagon in the pancreas was determined by immunoblotting analysis. Panels on the right show the quantification of the expression that was normalized to α-tubulin * denotes statistical difference *(p* < 0.05) and ** denotes significant difference (*p* < 0.01) from the control.

Binge ethanol exposure appeared to alter pancreatic function. Ethanol exposure significantly decreased glucose levels in the plasma (Figure 5A), but no difference in the plasma levels of insulin and glucagon (Data not shown). However, ethanol markedly enhanced plasma α-amylase activity (Figure 5B), and the expression levels in pancreatic tissues as indicated by IHC (Figure 5C) and immunoblotting analysis (Figure 5D). In addition, the increased expression of insulin in pancreatic tissues was found in ethanol group mice, but no difference in the level of glucagon between the control and ethanol group (Figure 5E).

### Binge ethanol exposure causes pancreatic inflammation

**Figure 6:**
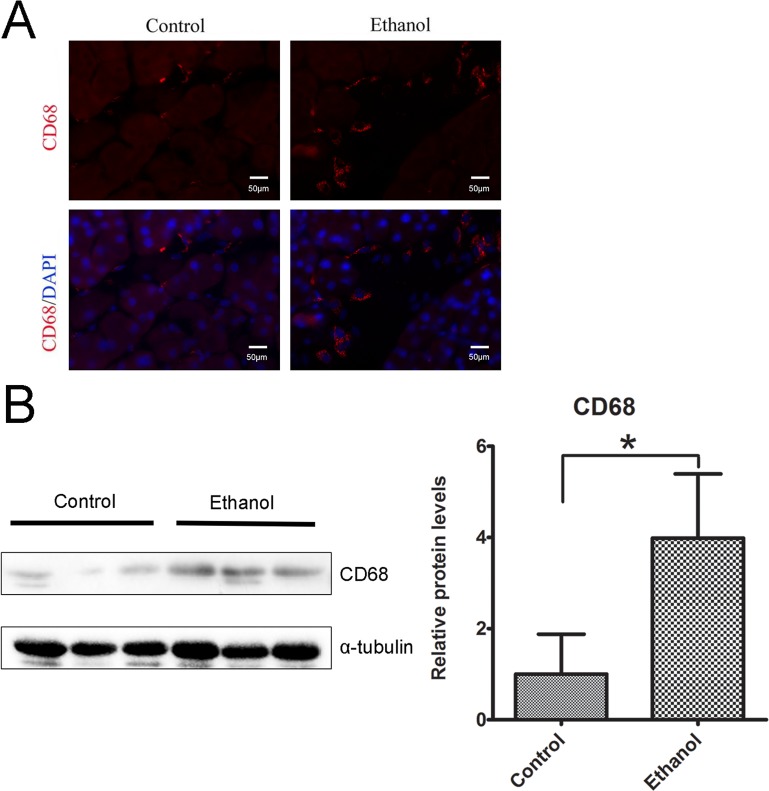
Binge ethanol exposure-induced macrophage infiltration **A.** The infiltration of macrophages in the pancreas was determine by CD68 immunofluorescent staining (Red). Nuclei were labeled with DAPI (blue). Bar = 50 μm (magnification of 40X). **B.** The relative protein expression of CD68 in the pancreas was determined by immunoblotting. The panel on the right is the quantification of CD68 expression that was normalized to α-tubulin. Each data point was the mean ± SEM of three independent experiments. * denotes statistical difference *(p* < 0.05).

**Figure 7:**
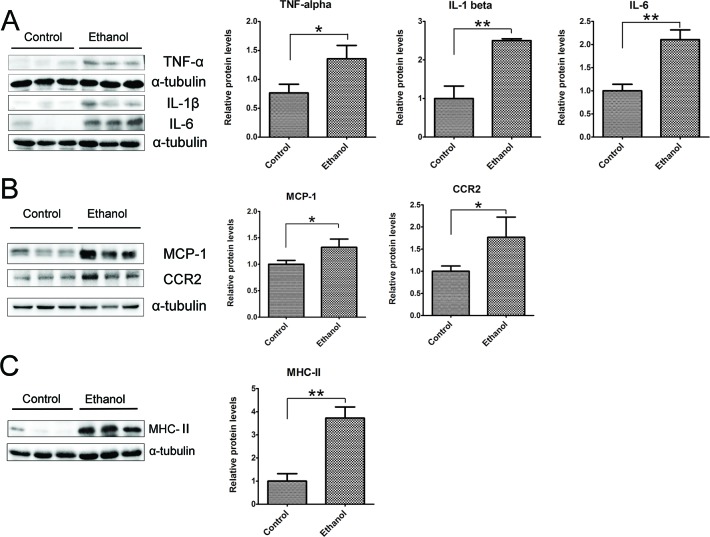
Binge ethanol exposure-induced pancreatic inflammation **A.** The expression of TNFα, IL-1β and IL-6 in the pancreas was determined by immunoblotting. **B.** The expression of MCP-1 and CCR2 in the pancreas was determined by immunoblotting. **C.** The expression of MHC-II in the pancreas was determined by immunoblotting. Each data point was the mean ± SEM of three independent experiments. Panels on the right show the quantification of the expression that was normalized to α-tubulin. * denotes statistical difference *(p* < 0.05) and ** denotes significant difference (*p* < 0.01) from the control.

To determine whether binge ethanol exposure caused pancreatic inflammation, we first examined macrophage infiltration in the pancreatic tissues. Binge ethanol exposure increased CD68 (a macrophage marker)-positive cells in the pancreatic tissues as shown by immunofluorescent staining (Figure 6A); this is confirmed by immunoblotting analysis which showed an increase in the expression level of CD68 (Figure 6B). We also examined the expression of cytokines and chemokines in the pancreatic tissues. As shown in Figures 7A and 7B, ethanol significantly increased the expression of TNFα, IL-1β, IL-6, monocyte chemoattractant protein (MCP-1) and its receptor CCR2 in the pancreatic tissues. Ethanol also increased the expression of major histocompatibility complex class II (MHC-II) which is expressed on antigen-presenting cells and involved in antigen presentation (Figure 7C).

### Binge ethanol exposure causes endoplasmic reticulum (ER) stress and oxidative stress

**Figure 8:**
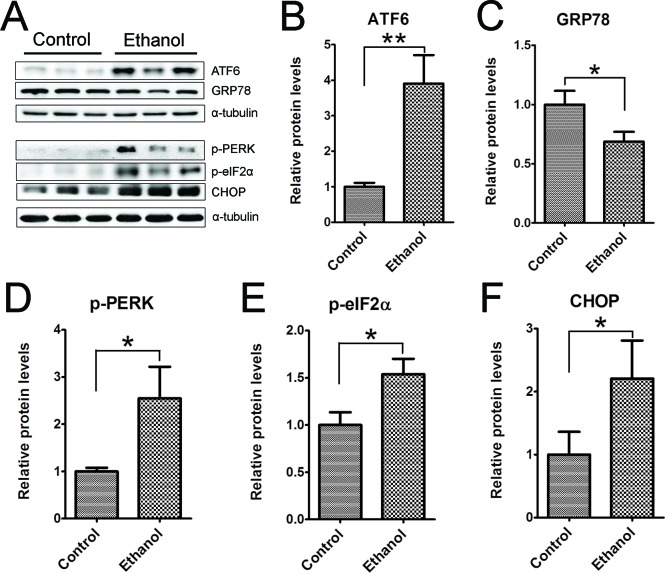
Binge ethanol exposure-induced endoplasmic reticulum (ER) stress in the pancreas Mice were exposed to binge ethanol for 10 days as described in the Materials and Methods. Six hours after last ethanol exposure, mice were euthanized and the pancreatic tissues were processed by immunoblotting analysis of ER stress markers **A.** The relative protein expression of ATF6 **B.**, GRP78/BIP **C.**, p-PERK **D.**, p-eIF2α **E.** and CHOP **F.** was quantified and normalized to α-tubulin. Each data point was the mean ± SEM of three independent experiments. * denotes statistical difference *(p* < 0.05) and ** denotes significant difference (*p* < 0.01) from the control.

To determine whether ethanol induced ER stress in the pancreas we examined the expression of a spectrum of proteins involved in the unfolded protein response (UPR) in the pancreatic tissues (Figure 8). Binge ethanol exposure significantly increased the expression of ATF6, CHOP, phosphorylated PERK (p-PERK) and p-eIF2α. However, ethanol significantly decreased the expression of GRP78.

**Figure 9:**
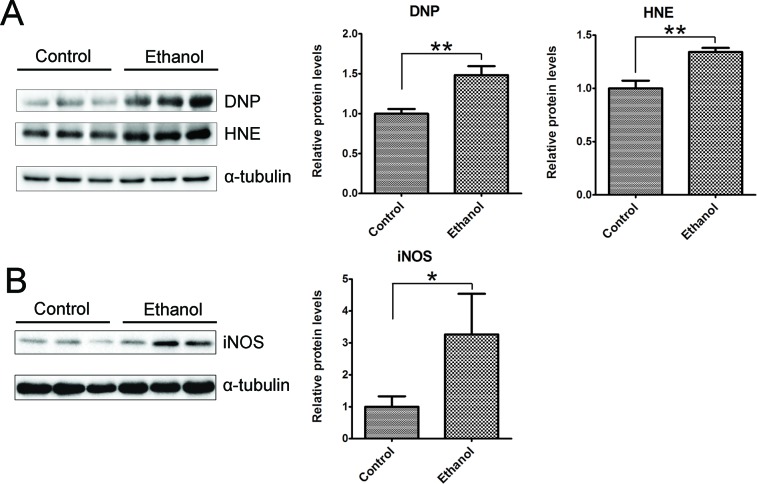
Binge ethanol exposure-induced oxidative stress in the pancreas After binge ethanol exposure as described above, mice were euthanized and pancreatic tissues were evaluated for oxidative stress. **A.** The levels of protein oxidation marker dinitrophenol (DNP) and lipid peroxidation marker 4-hydroxynonenal (HNE) were determined by immunoblotting. **B.** The expression of inducible nitric oxide synthase (iNOS) was determined by immunoblotting. Panels on the right show the quantification of the expression that was normalized to α-tubulin. Each data point was the mean ± SEM of three independent experiments. * denotes statistical difference *(p* < 0.05) and ** denotes significant difference (*p* < 0.01) from the control.

We then determined whether binge ethanol exposure caused oxidative stress in the pancreas. We examined protein oxidation and lipid peroxidation by assaying the protein carbonyl content with immunoblotting using an anti-dinitrophenol (DNP) antibody and lipid peroxidation byproduct using an anti-4-hydroxynonenal (4-HNE) antibody, respectively. As shown in Figure 9A, ethanol significantly increased the protein carbonyl content and 4-HNE. In addition, ethanol also increased the inducible nitric oxide synthase (iNOS) which can produce free radical nitric oxide (NO), inducing oxidative stress (Figure 9B).

## DISCUSSION

Alcohol abuse is one of the leading causes for AP [5, 6, 8]. The onset of AP may occur within the first 12 hours of the drinking episode or may be delayed by 2 to 3 days [18]. Overall, approximately 20% of patients with AP develop a severe case, and of those severe AP (SAP) cases 10-30% die [1]. Episodic heavy or binge drinkers tend to experience the severer and more complicated clinical courses of AP, resulting in higher total mortality and the incidence of complication. It has been suggested that binge drinking before the onset of the disease is a contributor to the aggravation of the first-attack SAP [18]. It remains controversial whether alcohol-related AP can develop in a normal pancreas or only in a pancreas already affected by chronic pancreatitis [10, 26]. We established an experimental model to investigate binge ethanol exposure-induced injury to the pancreas.

### Binge ethanol exposure and pancreatic injury

In this model, mice were exposed to a single episode of ethanol daily for 10 consecutive days. The oral gavage (5 g/kg; 25% w/v) produced high blood ethanol concentrations (BEC) (374-463 mg/dl) which mimics human binge drinking. This paradigm has been previously used to investigate binge ethanol exposure-induced neuroinflammation and neurodegeneration in the brain [27, 28]. The BEC achieved in the current study is consistent with that previously reported [27, 28].

We show here that this paradigm of binge ethanol exposure caused a spectrum of pancreatic tissue injury and inflammation. At histological levels, ethanol exposure reduces the size of acinar cells and the volume of acinar tissue while increasing the space between the acini, suggesting the occurrence of tissue edema and acinar atrophy. In ethanol-exposed tissues, some spindle-shaped stromal cells appear in the pericellular or intralobular areas of the pancreas and increased expression of vimentin is observed, which is usually indicative of the activation of pancreatic stellate cells [22]. Together, the observation points to the progression of fibrosis. At the cellular levels, ethanol causes apoptotic cell death which is evident by the increase in active caspase-3, caspase-8, cleaved PARP and cleaved CK-18. It appears that the increased CK-18 is located in the acini. Although histological analysis by H&E staining does not clearly demonstrate necrotic cell death, such as pyknotic cells showing plasma membrane rupture and dilatation of cytoplasmic organelles, a significant increase in the expression of HMGB1 in the acini of pancreas is observed by immunoblotting analysis and IHC (Figure 3). Increased HMGB1 in the pancreas is associated with pancreatic necrosis and frequently used to demonstrate necrotic cell death in pancreatitis patients and ethanol-induced injury to the pancreas [29]. This is consistent with previous reports that apoptosis and necrosis are the two forms of cell death observed in the exocrine pancreas in ethanol-promoted pancreatitis [8]. Ethanol-induced histological alteration and cell death are accompanied by an increase in cell proliferation in the acini (Figure 4), suggesting the regeneration following the injury. Ethanol induces pancreatic inflammation which is indicated by the increase of TNF-alpha, IL-1beta, IL-6, MCP-1 and CCR2 as well as the increase of CD68 positive macrophages in the pancreas. As a result, ethanol directly or indirectly alters the function of the pancreas which is indicated by the changes in both tissue and plasma levels of alpha-amylase, glucose and insulin. Experimental AP is defined by the development of relevant edema, inflammation and necrosis in the pancreatic tissue [10]. The ethanol-induced histological/pathological changes and inflammation reported in this study are characteristic of Experimental AP.

The pancreas contains both exocrine and endocrine components. The endocrine component comprising only 1-2% of the pancreas produces insulin and glucagon which are responsible for the regulation of glucose homeostasis. The exocrine component comprises the vast majority of the pancreas contains acinar, stellate and ductal cells. The acinar cells produce digestive enzymes and the ductal cells form a network that functions to deliver these enzymes into the duodenum. The pancreatic stellate cells synthesize and degrade extracellular matrix proteins and are activated during pancreatic injury [2]. Pancreatitis mainly results from the damages to the exocrine component, particularly the acinar cells [30]. In our study, ethanol-induced histological/cellular alterations are mainly observed in the exocrine component. However, a closer examination of the endocrine component is necessary to determine whether the binge ethanol exposure also affects the endocrine system as well.

Until our study, acute ethanol exposure alone has been unable to induce experimental alcoholic AP [10, 31]. For example, in a study where rats were acutely exposed to ethanol through either gavage or intravenous delivery, ethanol caused the impairment in pancreatic microcirculation and an increase of serum amylase levels without histological/morphological signs of experimental AP. The BEC of that study was 150-250 mg/dl which is much lower than ours (374-463 mg/dl). The ethanol exposure duration in this study (3-24 hours of ethanol exposure) is also much shorter than our study (10 days). Lack of histological/morphological signs of experimental AP in their paradigms is likely due to lower BEC and shorter duration of ethanol exposure. In a study using invasive delivery of ethanol of extremely high concentration of ethanol, ethanol (48%, 1 mL) was directly injected into the common biliary duct [32]. Twenty four hours after the injection, signs of experimental AP, such as edema, hemorrhage, inflammatory infiltration of neutrophils and mononuclear cells and necrosis, were observed [32]. However, the physiological relevance of this ethanol exposure paradigm is questionable.

Even long-term feeding of ethanol alone (one month or longer) causes minimal pancreatic tissue injury in animal models [8, 22, 33, 34]. Tsukamoto-French intragastric ethanol infusion or feeding with Lieber-DeCarli diet. The BEC achieved with these paradigms are usually around 200 mg/dl or less. Therefore, it is likely that the high peak BEC is an important determinant for ethanol-induced pancreatic injury.

### Mechanisms for ethanol-induced pancreatic injury

The pancreatic acinar cells have the ability to metabolize ethanol by both oxidative and non-oxidative pathways. The oxidative metabolism of ethanol is catalyzed by two enzymes: the cytosolic enzyme, alcohol dehydrogenase (ADH), and the microsomal enzyme, cytochrome P450 2E1 (CYP2E1). Ethanol metabolism by both of these enzymes generates acetaldehyde and reactive oxygen species [2, 23]. Non-oxidative metabolism of ethanol is carried out by a number of enzymes, the most important being the fatty acid ethyl ester synthases. Metabolism of ethanol by these enzymes generates fatty acid ethyl esters (FAEEs) which may also contribute to ethanol toxicity in the pancreas [2, 23]. It is unclear in our study whether the injury is mediated by ethanol directly or by its metabolites/by-products.

We show here that binge ethanol exposure induces endoplasmic reticulum (ER) stress and oxidative stress. The acinar cell of the exocrine pancreas has a highly developed ER system for the synthesis and secretion of digestive enzymes [35]. The ER regulates posttranslational protein processing and transport. The regulation requires optimal redox conditions and ion concentrations such as calcium for the ER enzymes to function properly. The disruption of this process results in the accumulation of unfolded or misfolded proteins in the ER lumen, triggering ER stress and inducing unfolded protein response (UPR) which are mediated by three transmembrane ER signaling proteins: pancreatic endoplasmic reticulum kinase (PERK), inositol-requiring enzyme 1 (IRE1) and activating transcription factor 6 (ATF6). If ER stress exceeds the capacity of UPR to clear the accumulation of unfolded or misfolded proteins in the ER lumen, cell death will occur. The ER of the acinar cell in the exocrine pancreas requires a robust UPR system considering the fact that its protein synthesis demands are the greatest of any tissue in the body [36].

It has been reported that long-term ethanol feeding (4-6 weeks) in mice and rats causes ER stress, which activates a UPR and increases XBP1 levels and activity [33]. In wild-type mice, however, this long-term ethanol exposure-induced pancreatic damage was very minor. XBP1 is an important regulator of UPR. In XBP-1 knock-out (*Xbp1*-*/*-) mice, ethanol feeding induces much more severe ER stress and pancreatic damage [33]. Therefore, it is likely that an adaptive UPR may protect against ethanol-induced damage to the exocrine pancreas by alleviating ER stress. In our system, it is unclear whether ER stress mediates ethanol-induced pancreatic damage. Further study using approaches to either inhibit or promote UPR will be necessary to offer more insight.

We also show that ethanol causes oxidative stress which is one of proposed mechanisms for ethanol-induced pancreatic damage [32, 37, 38]. There is considerable interaction between oxidative stress and ER stress [39]. Oxidative stress has been proposed as an important mechanism for ethanol-induced ER stress in multi-organ injury [39, 40]. Therefore, oxidative stress is a likely factor causing pancreatic ER stress in ethanol-exposed animals. In addition to oxidative stress, aberrant calcium signaling has also been considered an important factor in the initiation of pancreatic injury [2, 37]. Ethanol and its metabolites may disrupt calcium homeostasis in the pancreas [37, 41]. Disruption of calcium homeostasis may also cause ER stress which results in the damage to the pancreas as described above. In summary, the mechanisms underlying ethanol-induced pancreatic damage are complex and it is possibly the interaction between oxidative stress, ER stress and inflammation that contributes to the detrimental effects of ethanol on the pancreas.

## MATERIALS AND METHODS

### Materials

Reagents for the analysis for ethanol and glucose concentration were obtained from Analox instruments (London, UK). Rabbit anti-α-amylase, mouse anti-insulin, mouse anti-glucagon and mouse anti-tubulin antibodies were obtained from Sigma-Aldrich (St. Louis, MO). Mouse anti-M30 CytoDEATH (caspase cleavage product of cytokeratin 18) was obtained from Roche Life Science (Mannheim, Germany). Rabbit anti-p-eIF2α, rabbit anti-p-PERK, rabbit anti-cleaved caspase-3, rabbit anti-PARP, mouse anti-caspase-8, rabbit anti-Ki67, rabbit anti-HMGB1 and rabbit anti-Dinitrophenol (DNP) antibodies were obtained from Cell Signaling Technology (Danvers, MA). Rabbit anti-GRP78 antibody was obtained from Novus Biologicals (Littleton, CO). Mouse anti-CHOP antibody was obtained from Thermo Fisher Scientific (Rockford, IL). Rabbit anti- 4-Hydroxynonenal (HNE) antibody, rabbit anti-ATF6 and mouse HMG1 / HMGB1 ELISA kit were obtained from LifeSpan BioSciences (Seattle, WA). Mouse anti-iNOS/NOS II antibody was obtained from EMD Millipore (Billerica, MA). Rat anti-CD68 and rabbit anti-MCP-1 antibodies were obtained from AbD Serotec (Oxford, UK). Rabbit anti-CCR2 antibody was obtained from BioVision (Milpitas, CA). Rabbit anti-IL-1beta, rabbit anti-IL-6, and mouse anti-MHC-II antibodies and Amylase Assay kit were obtained from AbCam (Cambridge, MA). Mouse anti-vimentin antibody was obtained from BD pharmingen (San Diego, CA). HRP-conjugated anti-rabbit, anti-mouse, anti-goat and anti-rat secondary antibodies were purchased from GE Healthcare Life Sciences (Piscataway, NJ). Biotin-conjugated anti-rabbit secondary antibody, ABC kit and mounting media with DAPI were obtained from Vector Laboratories (Burlingame, CA). Alexa-488 conjugated anti-rabbit and Alexa-594 conjugated anti-rat antibodies were obtained from Life Technologies (Grand Island, NY). Liquid DAB substrate kit was obtained from Invitrogen (Carlsbad, CA). Ketamine/xylazine was obtained from Butler Schein Animal Health (Dublin, OH). Other chemicals and reagents were purchased either from Sigma-Aldrich or Life Technologies (Frederick, MD).

### Animal model

Male C57BL/6 mice (8 weeks old) were obtained from Jackson Laboratories (Bar Harbor, Maine) and maintained in the Division of Laboratory Animal Resources of the University of Kentucky Medical Center. Only male mice were used in this study because in humans, males are more susceptible to alcoholic AP [42]. All procedures were performed in accordance with the guidelines set by the National Institutes of Health (NIH) Guide for the Care and Use of Laboratory Animals and were approved by the Institutional Animal Care and Use Committee (IACUC) at the University of Kentucky. Animals were maintained in a 12 hour/12 hour light/dark cycle with temperature of 22±1°C and relative humidity of 60±5%, and received standard chow and water ad libitum. After one week of acclimation, mice were divided into an ethanol treatment group and a control group (*n* = 8 for each group). Mice were treated with ethanol (5 g/kg; 25% w/v) or water (equal volume) by gavage once daily for 10 days at 10:00 am. Six hours after final ethanol treatment, mice were euthanized and the pancreas was dissected and processed for histological and biochemical analyses.

### Determination of blood ethanol and glucose concentrations

At one hour following gavage on day 5 and day 10, mice were anesthetized by intraperitoneal (IP) injection of ketamine/xylazine and blood samples were taken *via* the retro-orbital sinus using a tube coated with K2EDTA (BD, Franklin Lakes, NJ). The plasma was obtained by centrifugation and 10 μl was used to measure blood ethanol and glucose concentration using an Analox AM 1 analyzer (Lunenburg, MA) as previously described [43]. The blood ethanol concentration (BEC) on day 5 and day 10 was 374 ± 22 mg/dl and 463 ± 25 mg/dl, respectively.

### Measurement of plasma amylase and HMGB1

The plasma was separated and stored at −80°C for ELISA assay. The α-amylase activity of plasma was assessed using the amylase assay kit from Abcam (Cambridge, UK) in accordance with the manufacturer's instructions. 25 μl of the plasma sample was diluted to 50 μl for each test, and the activity of α-amylase was recorded as mU/ml (nmol/min/ml). The plasma HMGB1 levels were detected by HMG1 / HMGB1 ELISA Kit obtained from LifeSpan BioSciences (Seattle, WA) according to the manufacturer's description.

### Tissue preparation and immunoblotting

Animals were anesthetized with intraperitoneal injection of ketamine/xylazine (100 mg/kg/10 mg/kg), and the pancreas was dissected and immediately frozen in dry ice and then stored in −80°C. The protein was extracted and subjected to immunoblotting analysis as previously described [44]. Briefly, tissues were homogenized in an ice cold lysis buffer containing 50mM Tris-HCl (pH 7.5), 150 mM NaCl, 1 mM EGTA, 0.5% NP-40, 0.25% SDS, 1 mM PMSF, 5 μg/ml leupeptin, and 5 μg/ml aprotinin. Homogenates were centrifuged at 20,000 g for 30 min at 4°C and the supernatant fraction was collected. After determining protein concentration, aliquots of the protein samples (30 μg) were separated on a SDS-polyacrylamide gel by electrophoresis. The separated proteins were transferred to nitrocellulose membranes. The membranes were blocked with either 5% BSA in 0.01 M PBS (pH 7.4) and 0.05% Tween-20 (TPBS) at room temperature for 1 hour. Subsequently, the membranes were probed with primary antibodies overnight at 4°C. After three washes (5 min each) in TPBS, the membranes were incubated with a secondary antibody conjugated to horseradish peroxidase. The immune complexes were detected by the enhanced chemiluminescence substrate (GE Healthcare, Chalfont, Buckinghamshire, UK). The density of immunoblotting was quantified with the software of Image lab 5.2 (Bio-Rad Laboratories, Hercules, CA).

### Immunohistochemistry, histological analysis and immunofluorescent staining

The procedure for immunohistochemistry (IHC) has been previously described with some modifications [44]. Briefly, animals were anesthetized by intraperitoneal (IP) injection of ketamine/xylazine and intracardially perfused with 0.01M PBS, and then by 4% paraformaldehyde in PBS (pH 7.4). The pancreatic tissues were removed, and post fixed in 4% paraformaldehyde for 24 hours and then transferred to 10%-30% sucrose in PBS until the tissues sunk to the bottom. The tissues were frozen in OCT compound and sectioned at the thickness of 15 μm using a Cryostat Microtone (Thermo Scientific). The sections were incubated in 0.3% H_2_O_2_/30% methanol in PBS for 20 min. After washing with PBS, the slides were blocked with 1% BSA and 0.5% TritonX-100 in PBS for 1 hour at room temperature. After blocking, the slides were treated with a rabbit anti-Ki67 antibody (1:400) overnight at 4°C. After washing with PBS, slides were incubated with biotin-conjugated goat anti-rabbit secondary antibody (1:800) for 1 hour at room temperature and followed by PBS washes. Avidin-biotin-peroxidase complex was prepared according to the manufacturer's instructions. After rinsing, the slides were developed in 0.05% 3,3′-Diaminobenzidine (Invitrogen) containing 0.003% H_2_O_2_ in PBS. The sections were then dehydrated through graded alcohol, and cleared with xylene and mounted with synthetic resin. The images were recorded using an Olympus BX51 microscope. Negative controls were performed by omitting the primary antibody. Ki67 positive cells were counted at 40X magnification. Twenty randomly selected sections covering at least 1,000 cells were counted. Four-five animals were analyzed for each group.

For histological analysis, the sections were stained with H&E reagent (Sigma-Aldrich). The percentage of area occupied by acini in total pancreatic tissue was calculated by an image analysis system (Image lab 5.2, Bio-Rad Laboratories, Hercules, CA) as described previously [22]. The tissue injury (necrosis and fibrosis) was analyzed as described previously [20, 21]. Twenty fields were randomly selected for the analysis. Three-four animals were analyzed for each group.

The procedure for immunofluorescent staining has been previously described with some modifications [45]. Briefly, the pancreatic sections were prepared at the thickness of 10 μm. After blocking with 1% BSA and 0.5% TritonX-100 in PBS for 1 hour at room temperature, the slides were incubated with a rabbit anti-α-amylase (1:400) or rat anti-CD68 (1:100) overnight at 4°C. After rinsing in PBS, the sections were incubated with Alexa Fluor 488-conjugated anti-rabbit or Alexa Fluor 594-conjugated anti-rat IgG in the dark at room temperature for 1 hour. After rinsing, the slides were covered with mounting media with DAPI and examined/recorded using a fluorescence microscope (IX81, Olympus). Negative controls were performed by omitting the primary antibody.

### Statistics

Quantitative data were presented as the means ± SEM. Differences between two groups were analyzed using *t* tests (nonparametric tests). Differences in which *p* was less than 0.05 were considered statistically significant.
